# Supplementary material for: Complexome profiling of the Chlamydomonas psb28 mutant reveals TEF5 as an early PSII assembly factor
Source: Plant Cell. 2025 Mar 17;37(6):koaf055. doi: 10.1093/plcell/koaf055 (PMC12164590; doi:10.1093/plcell/koaf055)
Supplement: koaf055_Supplementary_Data [file koaf055_supplementary_data.zip › psb28_interactive.zip!/plots/intensity/qualityPlots/peptides/CGL108_Cre12.g540500.t1.2.html]

### Cre12.g540500.t1.2

Trivial name: CGL108  
SeqCoverage [median]: 12.70  
Mass: 26.54 kDa  
  
 

| Sequence | MissedCleavage | Mass [Da] | wtIntensity | muIntensity | Score |
| --- | --- | --- | --- | --- | --- |
| FLQDVNSLR | 0 | 1090.58 | 575.77 | 0.00 | 46.43 |
| LVVSLQPK | 0 | 882.55 | 0.00 | 949.84 | 68.92 |
| SKPQEDFLDK | 1 | 1205.59 | 0.00 | 891.42 | 40.59 |
| SKPQEDFLDKTSR | 2 | 1549.77 | 1062.97 | 2959.12 | 49.31 |
|  |  |  | 1638.7 | 4800.4 |  |
